# ANLN and KDR Are Jointly Prognostic of Breast Cancer Survival and Can Be Modulated for Triple Negative Breast Cancer Control

**DOI:** 10.3389/fgene.2019.00790

**Published:** 2019-10-04

**Authors:** Xiaofeng Dai, Yi Mei, Xiao Chen, Dongyan Cai

**Affiliations:** ^1^Wuxi School of Medicine, Jiangnan University, Wuxi, China; ^2^School of Biotechnology, Jiangnan University, Wuxi, China; ^3^Department of Oncology, Affiliated Hospital of Jiangnan University, Wuxi, China

**Keywords:** ANLN, KDR, interaction, state transition, subtype, survival

## Abstract

**Purpose:** Kinase insert domain receptor (KDR) is the primary vascular endothelial growth factor receptor mediating survival, growth, and migration of endothelial cells and is expressed also in various tumor cells through autocrine production. The PI3K/Pten pathway is one of the downstream signalings affected by KDR activation and most commonly altered in breast cancer. Here, we investigate whether KDR expression is associated with members in PI3K/Pten signaling on the prognosis of breast cancer patients.

**Methods:** PI3K/Pten pathway components were defined by mapping The Cancer Genome Atlas (TCGA) protein data to the KEGG database complemented by literature searching, accounting for 36 proteins subject to the interaction analysis with KDR on breast cancer patient survival. The identified interaction gene pair was subjected to *in vitro* validation following functional analysis.

**Results:** Anillin (ANLN) was found to interact with KDR at translational and transcriptional levels using the public TCGA protein expression data and five gene expression datasets. Favorable prognosis corresponds to high protein but low gene expression of ANLN when KDR is highly expressed. Externally modulating cells toward low *ANLN* and high *KDR* gene expression was shown to transit triple negative cells toward a luminal-like state with increased level of ER and elevated sensitivity to Tamoxifen.

**Conclusion:** Our study proposes a two-gene panel prognostic of breast cancer survival and a novel therapeutic strategy for triple negative breast cancer control via transiting cancer cells towards a luminal-like state sensitive to established targeted therapy.

## Introduction

Vascular endothelial growth factor receptors (VEGFRs) are receptor tyrosine kinases mediating the survival, growth, and migration of endothelial cells through paracrine signaling (Deng et al., 2018). The downstream effects of VEGFR activation are mediated by a number of signaling cascades such as the mitogen-activated protein kinase and the PI3K/Pten pathways, where PI3K/Pten is frequently altered in breast cancers (Li et al., 2017). The intimate connections and regulatory relationships between VEGFR and PI3K/Pten signaling in tumors motivate us to investigate the joint prognostic value of VEGFR and components involved in the PI3K/Pten pathway on breast cancer clinical outcome. We conducted pair-wise interaction survival analysis between kinase insert domain receptor (KDR) [also named VEGFR2 and is the primary VEGFR (Takahashi and Shibuya, 2005)] and PI3K/Pten players at both transcriptional and translational levels using data retrieved from The Cancer Genome Atlas (TCGA), European Genome-Phenome Archive (METABRIC) (Curtis et al., 2012), and Gene Expression Omnibus database (Edgar et al., 2002), followed by a series of experimental validations. We demonstrate that low *ANLN* and high *KDR* gene expression is associated with favorable breast cancer outcome; externally forcing cancer cells to exhibit such a profile could transit cells from the triple negative to luminal-like phenotype and sensitize cells to Tamoxifen (Kumar et al., 2018) treatment due to possibly upregulated ER expression. Our results contribute in identifying a two-gene panel prognostic of breast cancer clinical outcome and propose a combined therapeutic strategy for triple negative breast cancer control.

## Materials and Methods

### Data

Data used in this study are summarized in **Supplementary Table 1**.

#### Protein Expression Data

The level 2 primary breast tumor reverse-phase protein microarrays data were retrieved from TCGA (http://cancergenome.nih.gov), which contains 385 samples. Super curve log2 values were linearized, median centered by the median across all samples, and normalized by the median across the entire panel of antibodies following the protocol (https://www.mdanderson.org/research/research-resources/core-facilities/functional-proteomics-rppa-core/faq.html).

#### Gene Expression Data

The level 3 primary breast tumor mRNA expression data were retrieved from TCGA, which includes 514 samples and 65 breast cancer death events. The mRNA data were produced using Agilent 244K Custom Gene Expression G4502A-07-3 platform, locally weighted scatterplot smoothing normalized followed by log2 transformation of the ratio between two channels.

The mRNA expression data from METABRIC (Curtis et al., 2012) were retrieved with permission, which include 1,293 samples and 295 breast cancer death events. The mRNA data were produced using Affymetrix SNP 6.0 and normalized using the quantile-based approach.

Three public datasets from GEO (Edgar et al., 2002), i.e., GSE6532 (Loi et al., 2007) and GSE22220 (Buffa et al., 2011), and GSE24450 (Muranen et al., 2011) were retrieved. GSE6532, including 87 samples (with 28 relapsed cases), was produced using Affymetrix Human Genome U133 Plus 2.0 Array and quantile normalized in robust multiarray analysis (Bolstad et al., 2003). GSE22220 was composed of 216 samples (including 82 distant relapsed events), produced using Illumina HumanRefSeq-8_V1 expression BeadChips, and normalized using the quantile-based approach. GSE24450 contains 183 primary breast tumors (39 breast cancers died of breast cancer or having distant metastasis), produced using Illumina HumanHT-12_V3 Expression BeadChips, and quantile normalized.

#### Histopathological Data

The histopathological data were retrieved from TCGA, which contains information on ER, PR, HER2, tumor size, nodal metastasis, and the tumor, node, and metastasis (TNM) stage (**Table 1**).

**Table 1:** Associations of the interaction between ANLN and KDR with histopathological parameters. The expression level, “high” or “low,” refers to that of ANLN and KDR, respectively, in the represented order. “ER,” “PR,” and “HER2” are cell receptors canonically used for breast cancer subtyping, “T” represents the size of the original tumor and whether it has invaded nearby tissue, “N” describes the nearby lymph nodes involved, “TNM stage” is an international standard for classifying the extent of spread of cancer based on “T,” “N,” and “M” (“M” describes distant metastasis). “Subtype” refers to PAM50 molecular subtyping, and ER-PR-HER2 histochemistry staining system was used to assess the subtyping status if PAM50 subtyping was not available; “LumAorB” means that PAM50 is “NA,” ER or PR is positive, HER2 is negative; “TNG” is short for triple negative group. Patients were analyzed by ANLN and KDR protein expression, with the number and percentage of patients in each category being summarized as “No.” and “(%).” Chi-squared test and 1,000 permutations of Monte Carlo simulations were conducted to assess the significance of associations of the two-gene interaction with each histopathological parameter.

ANLN:KDR	All		High:High		High:Low		Low:High		Low:Low		Chi-square	Monte Carlo
	No.	(%)	No.	(%)	No.	(%)	No.	(%)	No.	(%)	p	p
**ER**												
**−**	95	25.13%	28	28.57%	41	46.07%	7	6.09%	19	25.00%	1.91E−09	1.00E−04
**+**	283	74.87%	70	71.43%	48	53.93%	108	93.91%	57	75.00%		
**PR**												
**−**	148	39.05%	44	44.90%	48	53.93%	21	18.26%	35	45.45%	5.49E−07	1.00E−04
**+**	231	60.95%	54	55.10%	41	46.07%	94	81.74%	42	54.55%		
**HER2**												
**−**	203	75.46%	55	82.09%	44	72.13%	71	83.53%	33	58.93%	4.20E−03	5.20E−03
**+**	66	24.54%	12	17.91%	17	27.87%	14	16.47%	23	41.07%		
**T**												
**1**	90	23.50%	21	21.21%	16	18.18%	34	29.31%	19	23.75%	0.2776657	0.288471153
**2+**	293	76.50%	78	78.79%	72	81.82%	82	70.69%	61	76.25%		
**N**												
**0**	178	47.21%	53	54.08%	39	44.83%	53	47.32%	33	41.25%	0.3624281	0.360863914
**1+**	199	52.79%	45	45.92%	48	55.17%	59	52.68%	47	58.75%		
**TNM Stage**												
**1**	77	20.48%	17	17.35%	12	13.95%	32	28.07%	16	20.51%	0.0365787	0.0359964
**2**	213	56.65%	65	66.33%	46	53.49%	58	50.88%	44	56.41%		
**3**	86	22.87%	16	16.33%	28	32.56%	24	21.05%	18	23.08%		
**Subtype**												
**Basal**	28	7.37%	11	11.11%	10	11.36%	2	1.74%	5	6.41%	2.63E−07	1.00E−04
**Her2**	23	6.05%	4	4.04%	11	12.50%	1	0.87%	7	8.97%		
**LumA**	195	51.32%	50	50.51%	36	40.91%	77	66.96%	32	41.03%		
**LumAorB**	22	5.79%	10	10.10%	2	2.27%	6	5.22%	4	5.13%		
**LumB**	76	20.00%	14	14.14%	12	13.64%	27	23.48%	23	29.49%		
**TNG**	36	9.47%	10	10.10%	17	19.32%	2	1.74%	7	8.97%		

### Computational Methods

#### Expression Interaction Survival Analysis

The primary players of the PI3K/Pten pathway were defined using Kyoto Encyclopedia of Genes and Genomes (KEGG) (Kanehisa and Goto, 2000) supplemented by relevant literatures (Suzuki et al., 2005; Brouxhon et al., 2013; Quann et al., 2013; Thuma and Zoller, 2013). We first conducted survival analysis on pair-wise interactions at the translational level. In total, there were 142 antibodies available in TCGA, representing 114 unique proteins, among which 31 were involved in the PI3K/Pten pathway. These 31 genes plus 5 reported players of the PI3K/Pten pathway (Suzuki et al., 2005; Brouxhon et al., 2013; Quann et al., 2013) constitute the gene panel used in the interaction analysis (**Supplementary Table 2**, **Supplementary Figure 1**). Significant interactions at the translational level were selected for analysis at the transcriptional level following the same analytical procedure.

While TCGA data were used at the translational level, five datasets (TCGA, METABRIC, GSE6532, GSE22220, and GSE24450) were used at the transcriptional level. Anillin (ANLN) and KDR expressions were split into high and low levels at the splitting point optimized by grid searching (Barto, 1985). Binarized data were fitted into a Cox regression model, which include both the effect of each component and the interaction. In addition, a model without the interaction term was built for each pair. The *p* value from the chi-square test of the likelihood ratio between the model including the interaction term and the one without was used to assess the significance of the interaction. Kaplan–Meir plots were drawn to visualize the interactive effect.

Meta-analysis was applied in the analysis at the transcriptional level using the “metagen” function from the “meta” R package to assess the combined effect of the five datasets. The meta *p* value from the Fisher method (Fisher, 1932) was used to assess the significance of the interaction term. Stratified analysis, i.e., the survival was analyzed for one gene as stratified by the expression of the other, was conducted at both the protein and gene expression levels using the same statistical assessment methods.

Different death events were available in different datasets, i.e., 15-year breast cancer specific death in METABRIC, 10-year overall survival in TCGA data, 15-year relapse free survival in GSE6532, 10-year relapse free survival in GSE22220 data, and 10-year breast cancer specific death in GSE24450.

#### Histopathological Association Analysis

Samples were binarized into high and low expression of ANLN and KDR. The associations between tumors with different protein expressions of ANLN and KDR, and histopathological markers including ER, PR, HER2, T, N, TNM stage, and subtype classification were analyzed separately. The statistical significance was assessed by chi-square test and Monte Carlo simulation on 10,000 permutations in R.

### Experimental Materials

#### Cell Culture

One human normal mammary epithelial cell line (MCF10A), one luminal cell line (MCF7), and two triple negative breast cancer cell lines (MDAMB231 and SUM159PT) were included in the experiment. Cells were bought from the American Type Culture Collection, with mycoplasma tested and verified by sequencing.

MCF10A cells were cultured in Dulbecco’s modified eagle medium (DMEM)/F12 (Gibco) supplemented with 5% charcoal-stripped horse serum (Gibco), 10 µg/ml insulin (PeproTech), 20 ng/ml epithelia growth factor (PeproTech), and 1.4 × 10^−6^ mol/l hydrocortisone (PeproTech). MCF7 and MDAMB231 cells were cultured in DMEM supplemented with 10% fetal bovine serum (Gibco). SUM159PT cells were cultured in F12 (Gibco) supplemented with 5% fetal bovine serum (Gibco), 20 μg/ml insulin (PeproTech), 1% HEPES (PeproTech), 2.8 × 10^−6^ mol/l hydrocortisone (PeproTech). Assay ready cells were prepared by culturing cells in a large batch and aliquoting them into ampules that were kept in liquid nitrogen in solution containing 90% fetal bovine serum and 10% dimethyl sulfoxide. Immediately prior to transfection, cells were thawed and washed with culture medium, and cell number was counted using a hemocytometer (Thermo).

### Experimental Protocols

#### Cell Transfection

1 ×10^6^ cells per well were added in 2 ml of culture medium and transferred to black clear bottom tissue-culture treated six-well plates (Nalgene #167018). Cells were incubated overnight and achieved 70–80% confluence before transfection. Medium was replaced by 2 ml serum-free medium before transfection. One hundred microliter Optimem medium (Gibco) containing 1 μg sgRNA plasmids (sgRNAs were listed in **Supplementary Table 3**) and 1 μg dCas9-synergistic activation mediator (SAM) plasmids were added to 100 μl Optimem medium containing 6 μl lipo2000 transfection reagent per well and mixed for 15–20 min prior to transfection. The mixture was transferred to a six-well plate and incubated at 37°C for 5–8 h in the presence of 5% CO_2_ (HERA Cell 150i, Thermo Scientific). Serum-free medium was replaced by 2 ml medium containing 10% serum. Cells were incubated at 37°C for 24 h and then subjected to stable clone selection under 4-μl 200 mg/ml G418 and 5-μl 0.1 mg/ml puromycin pressure for 2 months.

#### qPCR Assay

After transfection, cells were collected and extracted for total RNA using TRIzol reagent (TianGen) at 3 days after transfection. The cDNA was synthesized using PrimeScript RT reverse transcriptase (Takara). Primers for quantitative reverse transcription PCR (qRT-PCR) are listed in **Supplementary Table 4**. The absorbance value was recorded at the extension stage. The relative expression level was calculated using the 2^−△△Ct^ methods. All qRT-PCR experiments were performed using ABI Step one plus Real-Time PCR System (ABI) following Takara protocol.

#### Proliferation Assay

Eight thousand cells per well were added in 100 μl of culture medium and transferred to black clear bottom tissue-culture treated 96-well plates (Nalgene #167008). Cells were incubated overnight and achieved 70–80% confluence before transfection, cells transfection as described above. For cell proliferation measurement, 10 μl per well of CKK-8 (Dojindo) was added, and absorbance was detected using EZ Read 800 microplate Reader (Biochrom) after cell incubation at 37ºC for 2 h.

#### Invasion Assay

After transfection, cells were incubated until they form confluent monolayers. Wounds were made using a pipette tip, and photographs were taken immediately (0 h), 12, 24, and 36 h after wounding. Distance change between the two edges of wounded area due to cell migration was measured and computed at each time point. Results were presented as the migration rate.

Student’s *t* test was computed using R to evaluate the statistical significance on cell migration, and *p* values were computed as the two-tailed probability at 95% confidence from a standard normal distribution.

#### Flow Cytometry Assay

The proportion of cancer stem cell was assessed by FACSCalibur flow cytometer (BD). Cultured cells were washed twice with phosphate-buffered saline (PBS) and then harvested using trypsin. Detached cells were washed once in PBS and stained using ALDEFLUOR™ kit (STEMCELL Technologies) at the room temperature (RT) in the darkness for 30 min. Labeled cells were washed and fixed in PBS and analyzed using flow cytometer.

#### Western Blot Assay

Cultured cells were washed twice using ice-cold PBS and lysed in radioimmunoprecipitation assay lysis buffer supplemented with protease inhibitors for 5 min on ice and centrifuged at 12,000g for 10 min before supernatants collection. The protein concentration was estimated using the BCA Protein Assay Kit (Tiangen). Proteins (50 μg) per lane were resolved by sodium dodecyl sulfate polyacrylamide gel electrophoresis and transferred to polyvinylidene fluoride membrane. After blocking with 5% nonfat dried milk powder in Tris-buffered saline plus Tween-20 buffer, the membrane was incubated using the appropriate primary Abs (Proteintech) at 4°C overnight followed by secondary Abs (Proteintech) for 2 h at RT. Ab binding was visualized by developing the blot using enhanced chemiluminescence reagent. The bands were visualized using OmegaLumG (UVP) followed by analysis using the Image J software. Western blot was performed after 72 h of construct transfection.

#### Drug Response Assay

MCF10A, MCF7, MDAMB231, and AdKu (*ANLN* downregulation and *KDR* upregulation) cells were used in the experiment. Eight Tamoxifen concentrations (1, 10, 25, 100, 250, 1,000, 2,500, and 10,000 nM) with six replicates were designed. Also included in each plate were the negative control and drug-free negative control at each drug concentration with six replicates. Tamoxifen (Sigma) was added to cells after they form confluent monolayers. Ten microliters per well of CKK-8 was added 48 h after adding Tamoxifen, and absorbance was detected using an EZ Read 800 microplate reader after cell incubation at 37ºC for 2 h. The dose–response curve of Tamoxifen treatment and IC50 values were obtained for each siRNA in each cell line using the “drc” package in R, where a four-parameter log logistic model (LL.4) was used for data fitting. Statistical significance on IC50 alteration was evaluated by Student’s *t* test using R.

#### Mouse *In Vivo* Study

1 × 10^6^ MDAMB231 and AdKu-231 cells suspended in PBS were injected subcutaneously to six female BALB/c mice aged 4–6 weeks with the average weight of 20 ± 5 g, respectively. Mice were divided into two groups, i.e., MDMA231 group, AdKu-231 group, depending on the tumor cells subcutaneously injected, and each group included four mice by design. Tumor volume was calculated using Equation (1)

(1) $$V=\frac{\pi \times L\times W^{2}}{6}$$

where “*V*,” “*L*,” and “*W*” each represents volume, the largest diameter, and smallest diameter of the tumor, respectively.

Tumor growth measuring started when tumor lesion appeared and recorded every 3 days. Mice were killed at the 24th day after the initial appearance of tumor lesions.

## Results

### Opposite Interactions Between ANLN and KDR at Translational and Transcriptional Levels

Among the 36 proteins being analyzed (**Supplementary Table 2**, **Supplementary Figure 1**), anillin (encoded by *ANLN*) was found to interact with KDR (also named VEGFR2), and such an interaction affected breast cancer survival with statistical significance at both the translational (**Supplementary Figure 2**, 51 and 44% were optimized for ANLN and KDR binarization, respectively) and transcriptional (**Supplementary Figure 3**, 51 and 32% were optimized for *ANLN* and *KDR* binarization, respectively) levels. Interactions between ANLN and KDR were confirmed by fitting the Cox regression model, where the fitness significantly improved when the interaction term was included at both translational (*p* = 0.006) and transcriptional (meta-analysis from five public datasets *p* = 0.024) levels (**Table 2**). No significant univariate clinical association was observed at the translational level for neither protein (**Supplementary Figure 2**). At the transcriptional level, *ANLN* had an independent main effect that was exemplified by *KDR* overexpression (**Supplementary Figure 3**), i.e., Fisher meta-analysis *p* value for *ANLN* was 8.07e−11 and became 3.59e−11 when *KDR* expression was high in the stratified analysis.

**Table 2:** Statistics of the model including the interactions between ANLN and KDR at the expression levels. “GEX” and “PEX” each represents the gene expression and protein expression, respectively. The expression level, “high” or “low,” each refers to that of ANLN and KDR, respectively, in the presented order. The 51 and 44% were (optimized using TCGA PEX data) used as the splitting point for binarizing ANLN and KDR PEX data, respectively; and 51 and 32% (optimized using METABRIC GEX data) were used as the splitting points for GEX data binarization, accordingly. “HR” and “95%CI” are the hazard ratio and 95% confidence interval ([low, high]) for each pair, respectively. The *p* value for the interaction term (p_inter) comes from the chi-square test, which shows the significance of the improvement of the model including the interaction term as compared with the model without interactions. “Meta-analysis” is conducted for GEX data, the meta-analysis *p* value (fixed-effects model given that no heterogeneity was detected) for each genotype combination is obtained using “metagen” from R package “meta,” and the meta-analysis for the interaction term is obtained using the Fisher’s method from the *p* values (p_inter).

Analysis	Data	ANLN:KDR	High:Low	Low:High	High:High	p_inter
PEX	TCGA	HR	2.5	***3.16***	0.16	0.0061
		95%CI	[0.88,7.10]	***[1.16,8.64]***	[0.04,0.63]	
GEX	METABRIC	HR	1.33	*0.88*	1.61	0.0692
		95%CI	[0.87,2.04]	*[0.60,1.31]*	[0.96,2.68]	
	TCGA	HR	0.87	*0.92*	1.42	0.5105
		95%CI	[0.38,2.02]	*[0.43,1.97]*	[0.50,4.02]	
	GSE6532	HR	0.58	*0.21*	10.29	0.0044
		95%CI	[0.18,1.91]	*[0.06,0.75]*	[1.99,53.18]	
	GSE22220	HR	2.55	*0.92*	1.22	0.7063
		95%CI	[1.08,5.99]	*[0.39,2.18]*	[0.44,3.39]	
	GSE24450	HR	3.39	*3.19*	0.41	0.2951
		95%CI	[0.68,16.80]	*[0.72,14.16]*	[0.07,2.36]	
Meta	GEX datasets	p	0.0779	*0.365*	**0.0244**	0.0235
		Method	Metagen	**Metagen**	Metagen	Fisher

**Figure 1:**
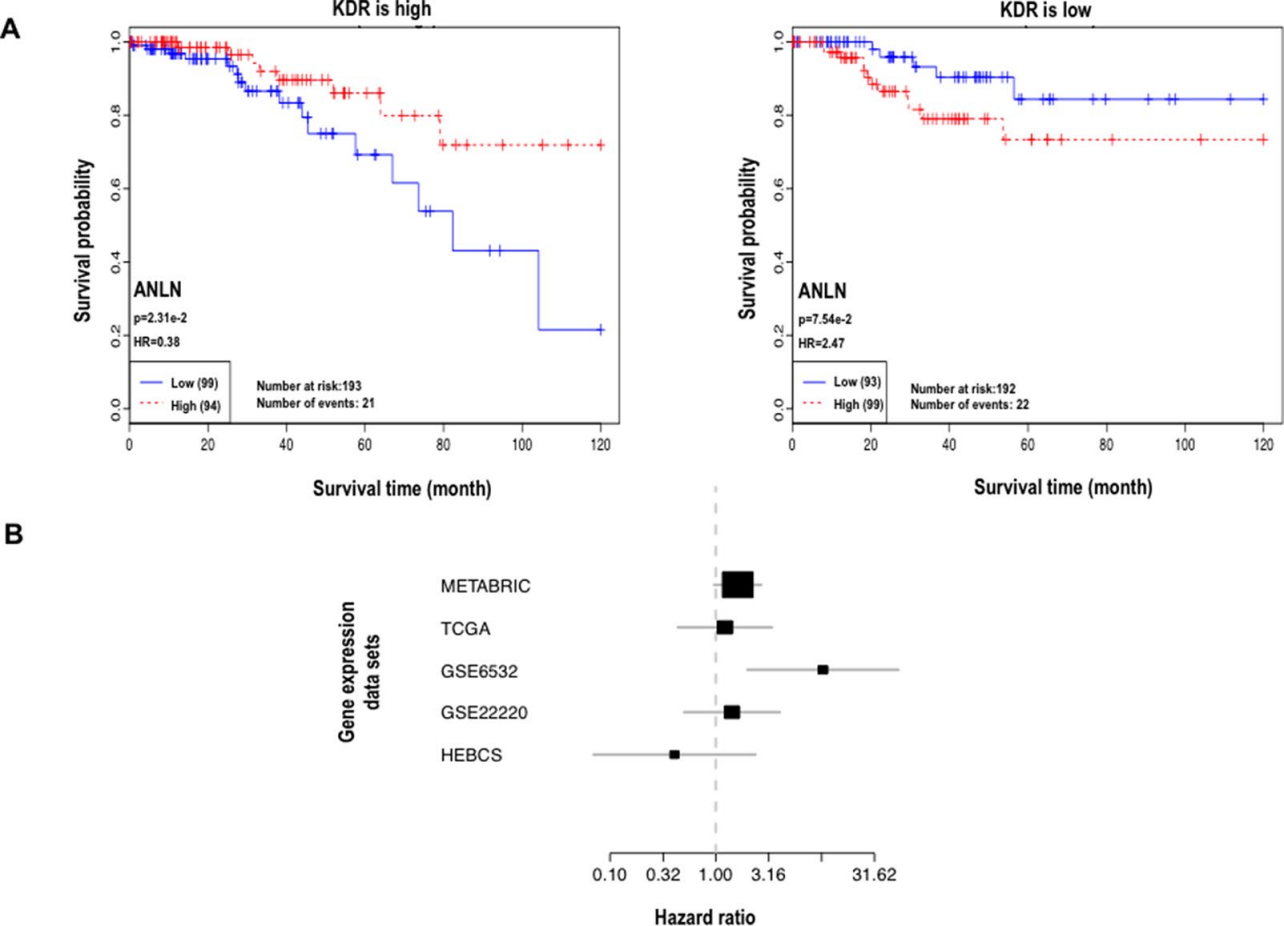
Analysis of interactions between ANLN and KDR. **(A)** Kaplan–Meier plot at the protein expression level. **(B)** Forest plot at the gene expression level. In the forest plot, each line represents the confidence interval of a study, where a longer line represents a smaller dataset; each black box represents a point estimation, where a larger area represents a higher weight the dataset contributes to the meta-analysis.

Interestingly, concomitant low ANLN and high KDR protein expression was associated with poor clinical outcome (HR = 3.16) but conveyed protective effect (HR < 1 for four out of five datasets) at the transcriptional level (**Table 2**, **Figure 1**, **Supplementary Figures 2** and **3**). In other words, low *ANLN* and high *KDR* gene expression shared the same clinical association with concomitant overexpression of both proteins, which was associated with favorable clinical outcome; concomitant high levels of both *ANLN* and *KDR* expression shared the same clinical outcome with patients having low ANLN and high KDR expression, which was associated with poor clinical outcome (**Figure 1**, **Table 2**).

**Figure 2:**
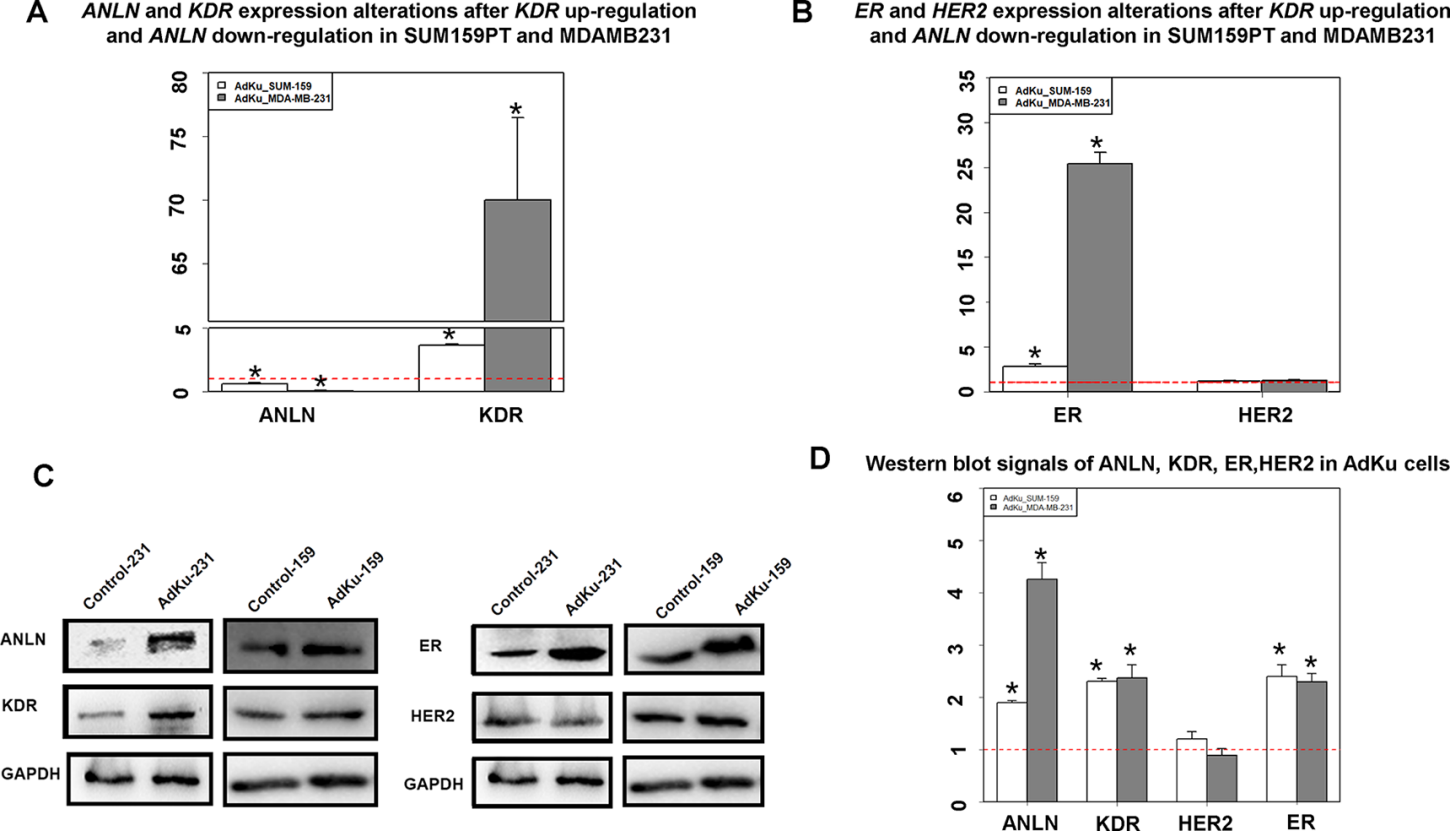
Expression of KDR, ANLN, ER, and HER2 in AdKu cells derived from triple negative breast cancer cells. **(A)** Expression of *KDR* and *ANLN* at the transcriptional level. **(B)** Expression of *ER* and *HER2* at the transcriptional level. **(C)** Expression of ANLN, KDR, ER, and HER2 at the translational level. **(D)** Western blot signaling intensities normalized by that of GAPDH for ANLN, KDR, ER, and HER2 in AdKu cells. * represents statistical significance (*p* < 0.05) Student’s *t* test. The red dotted line represents the expression level where no external modulation was done. MDAMB231 and SUM159PT cells were used to derive AdKu cells. The red dotted line represents the expression level where no external modulation was done.

We constructed two cell lines, namely, AdKu-231 and AdKu-159, with low *ANLN* and high *KDR* gene expression (**Figure 2A**). *ANLN* expression was significantly reduced (*p* = 0.008 for AdKu-231, *p* = 0.002 for AdKu-159) and that of *KDR* was significantly upregulated (*p* = 0.004 for AdKu-231, *p* = 0.005 for AdKu-159) in AdKu cells (**Figure 2A**). Western blotting showed concomitant overexpression of both proteins in both AdKu cells (**Figure 2C**). These results suggest that the observed opposite clinical associations at the translational and transcriptional levels lie in the reverse expression of ANLN at both gene and protein expression levels.

### Low ANLN and High KDR Gene Expression Is Associated With Less Malignant Breast Cancer Cell Features

**Figure 3:**
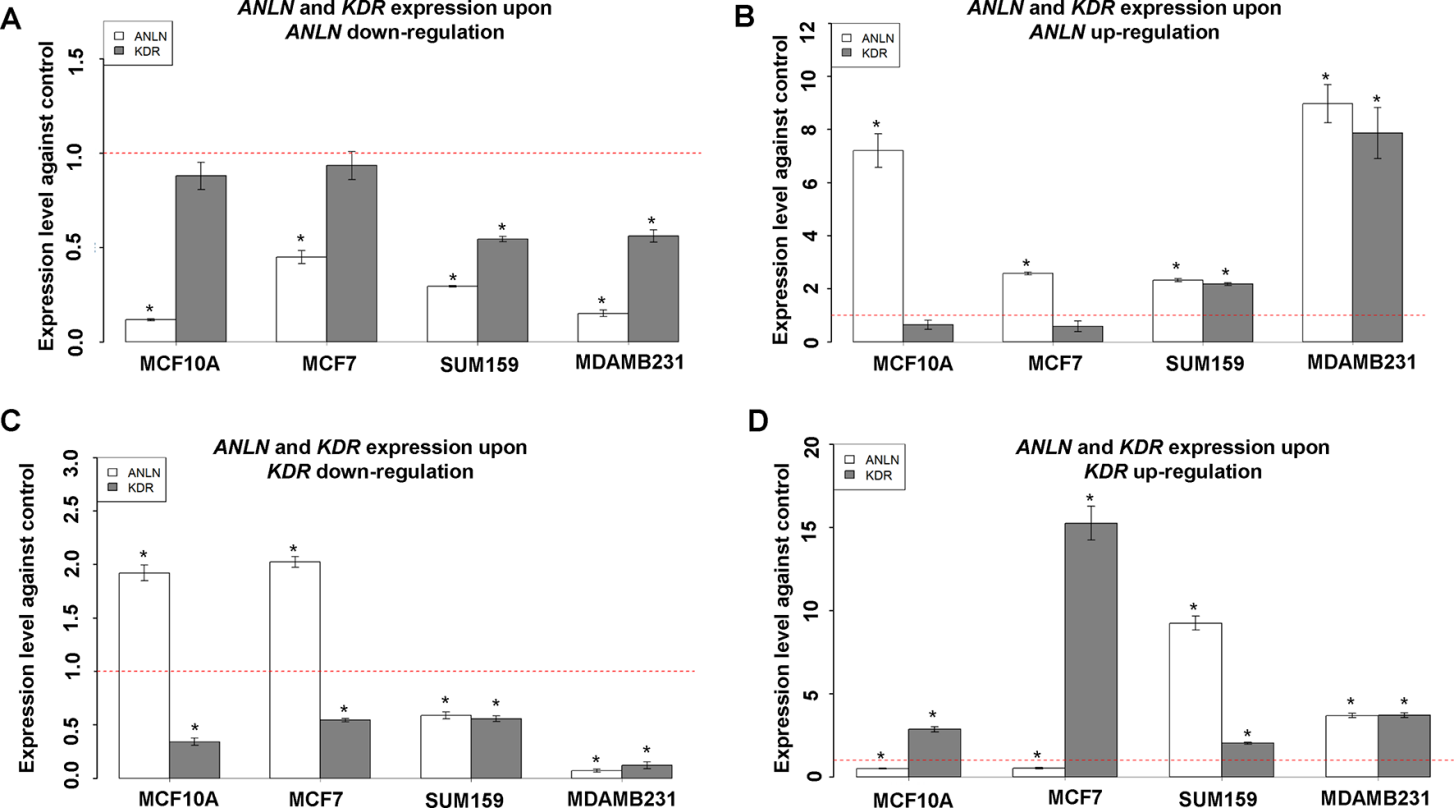
Interactions between *ANLN* and *KDR* in different breast cancer cells. **(A)** *KDR* gene expression after downregulating *ANLN* in each cell line. **(B)** *KDR* gene expression after upregulating *ANLN* in each cell line. **(C)** *ANLN* gene expression after downregulating *KDR* in each cell line. **(D)** *ANLN* gene expression after upregulating *ANLN* in each cell line. Bars represent mean ± SD from at least three independent experiments. * represents statistical significance (*p* < 0.05) from Student’s *t* test. The red dotted line represents the expression level where no external modulation was done. SUM159 and MDAMB231 are triple negative breast cancer cells, MCF7 is a luminal breast cancer cell line, and MCF10A represents normal breast epithelial cells.

*KDR* and *ANLN* were positively correlated at the transcriptional level when *ANLN* gene expression was perturbed in triple negative breast cancer cell lines SUM159PT and MDAMB231 (**Figures 3A, B**). In brief, *KDR* gene expression was significantly reduced (*p* = 5.54e−4 in SUM159PT, *p* = 0.010 in MDAMB231) once ANLN was effectively downregulated (*p* values were 0.001 and 3.53e−4, respectively, in SUM159PT and MDAMB231). When *ANLN* was sufficiently overexpressed (*p* values for upregulating *ANLN* were 2.00e−4 and 3.81e−4 in SUM159PT and MDAMB231, respectively), *KDR* expression increased with statistical significance (*p* = 5.12e−4 in SUM159PT, *p* = 0.002 in MDAMB231). Similarly, the expression of both genes was positively correlated when *KDR* was modulated in triple negative breast cancer cells (**Figures 3C, D**). That is, *ANLN* expression was significantly altered in the consistent direction with *KDR* (*p* = 0.010 for downregulation in SUM159PT, *p* = 2.41e−4 for downregulation in MDAMB231, *p* = 4.36e−5 for upregulation in SUM159PT, *p* = 4.72e−5 for upregulation in MDAMB231) when *KDR* expression was effectively down- and upregulated (*p* = 0.004 and *p* = 7.60e−4 for downregulation in SUM159PT and MDAMB231, respectively; *p* = 9.41e−4 and *p* = 9.14e−5 for upregulation in SUM159PT and MDAMB231, respectively).

We did not observe any significant alteration on *KDR* gene expression when modulating that of *ANLN* in the luminal breast cancer cell line MCF7 and normal breast cell line MCF10A (**Figures 3A, B**). *ANLN* gene expression was significantly modulated both up- and downwards (*p* values for downregulation were 2.49e−4 and 1.81e−4 in MCF7 and MCF10A, for upregulation were 1.33e−5 and 6.14e−4 in MCF7 and MCF10A, respectively), and no significant alteration was observed for *KDR* gene expression. However, we observed significant mutual suppression between *ANLN* and *KDR* gene expression in the luminal cell line MCF7 and normal breast cells MCF10A (**Figures 3C, D**). That is, *ANLN* was significantly downregulated (*p* = 8.41e−4 in MCF7 and *p* = 0.002 in MCF10A) when *KDR* was upward modulated (*p* = 1.53e−4 in MCF7, *p* = 3.97e-4 in MCF10A), and significantly upregulated (*p* = 5.82e−5 in MCF7 and *p* = 7.36e−4 in MCF10A) when *KDR* was downward modulated (*p* = 0.008 in MCF7, *p* = 7.72e−4 in MCF10A).

### Modulated Cells With Low ANLN and High KDR Gene Expression Exhibit Less Malignant Cancer Features

**Figure 4:**
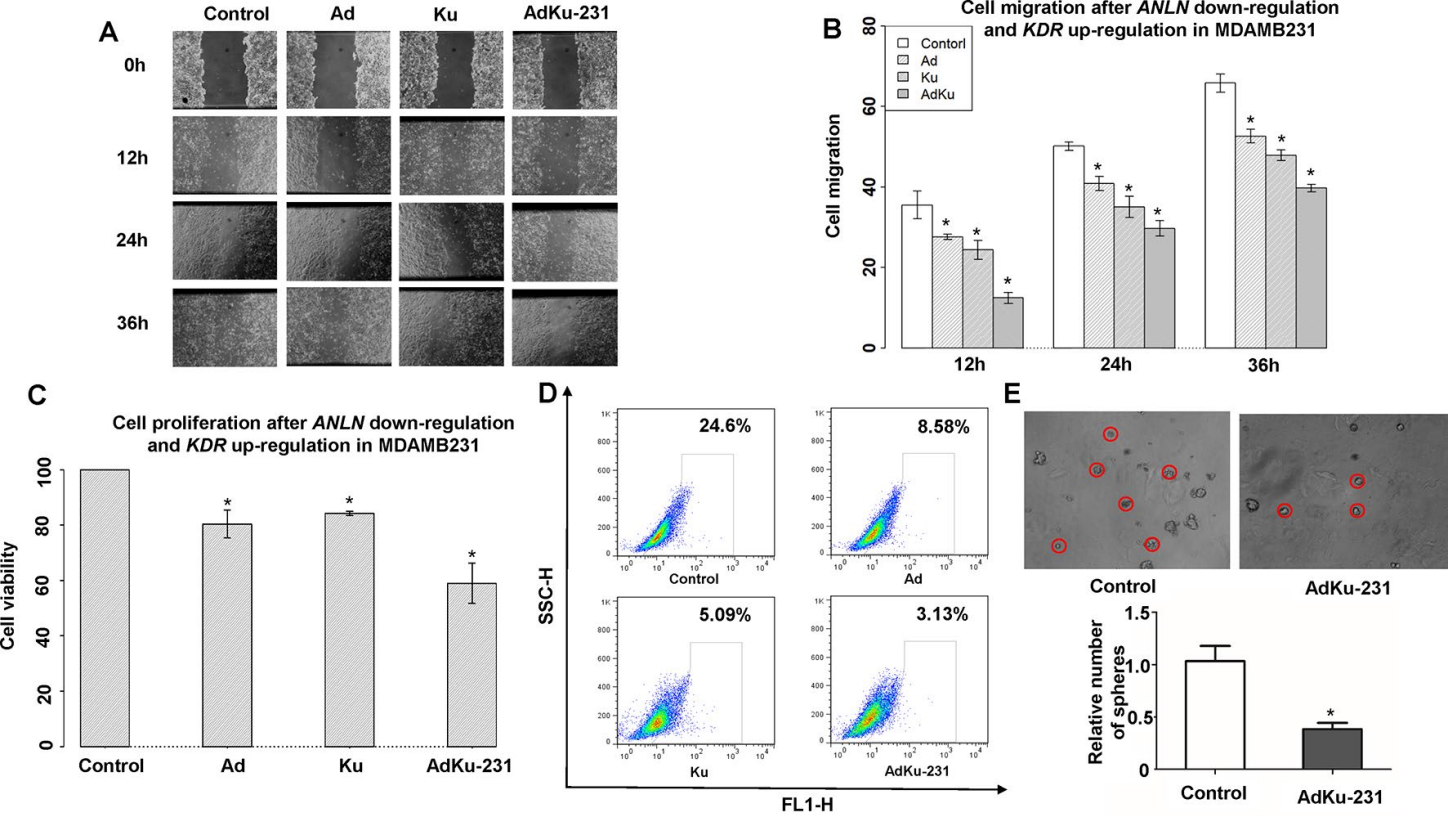
Cell morphological alterations in the stable AdKu cells derived from MDAMB231. Comparisons on the **(A)** images and **(B)** measured areas of cell migration, **(C)** cell proliferation, **(D)** stem cell percentage, and **(E)** cell self-renew ability. “Ad” represents the stable cell line with reduced ANLN gene expression, “Ku” represents the stable cell line with increased KDR gene expression, and “AdKu-231” means both are regulated. * represents statistical significance (*p* < 0.05) from Student’s *t* test.

We constructed a stable cell line, AdKu-231, with reduced *ANLN* and increased *KDR* gene expression from the triple negative breast cancer cell line MDAMB231 using the Crispr technique (sgRNAs are listed in **Supplementary Table 3**). *ANLN* and *KDR* were effectively modulated (*p* = 0.008 for knocking down *ANLN* and *p* = 0.004 for upregulating *KDR*, **Figure 2A**). The migration of AdKu-231 cells was significantly recessed as measured at 12 (*p* = 4.28e−4, 6.40e−5, 0.0017 as compared with MDAMB231, Ad, Ku), 24 (*p* = 8.71e−5, 0.002, 0.046 as compared with MDAMB231, Ad, Ku), and 36 (*p* = 5.13e−5, 3.36e−4, 0.001 as compared with MDAMB231, Ad, Ku) hours (**Figures 4A, B**). The growth of AdKu-231 cells was significantly reduced as compared with MDAMB231 (*p* = 1.91e−5), Ad (*p* = 8.99e−05), and Ku (*p* = 2.80e−4) cells (**Figure 4C**). The percentage of cancer stem cells was considerably reduced from 24.6% in MDAMB231 to 8.58% in Ad cells, to 5.09% in Ku cells, and to 3.13% in AdKu-231 cells (**Figure 4D**), and the relative number of spheres was reduced to 38% in AdKu-231 cells as compared with the control (*p* = 0.009, **Figure 4E**).

ER expression was significantly elevated in AdKu-231 cells, with *p* = 0.006 and *p* = 0.007, respectively, at the transcriptional and translational levels as compared with MDAMB231 cells (**Figures 2B, D**). Similar expression profiles were observed in AdKu-159 cells (**Figures 2B, D**). Histopathological association analysis revealed that ER status was significantly affected by the protein expression of ANLN and KDR, with the *p* value from chi-square test being 1.91e−09 and the *p* value from 1,000 permutations of Monte Carlo simulation being 1e−04. All three primary cell surface receptors used for breast cancer subtyping (ER, PR, and HER2) were significantly associated with ANLN and KDR expression (**Table 1**), suggesting that the synergistic effect of ANLN and KDR can affect cells’ transition from the triple negative to the luminal-like phenotype.

**Figure 5:**
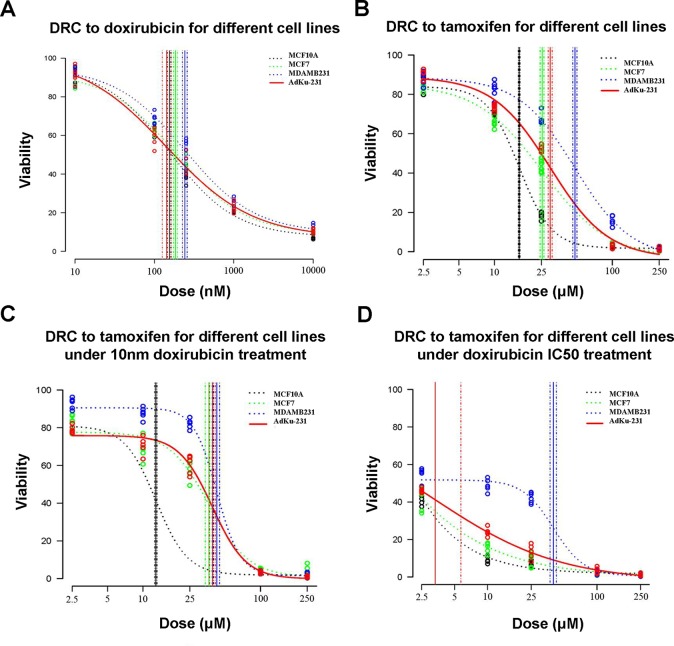
Comparison on cell viabilities in response to Tamoxifen, Doxirubicin, and combined use of Tamoxifen and Doxirubicin among different cell lines. Drug response curves under the treatment of **(A)** Tamoxifen, **(B)** Doxirubicin, **(C)** combined used of Tamoxifen and 10 nm Doxirubicin, and **(D)** combined use of Tamoxifen and IC50 Doxirubicin. AdKu-231 was used in this figure.

AdKu-231 cells show increased sensitivity to Tamoxifen, a commercialized drug-targeting ER-positive tumors. IC50 of AdKu-231 cells (29.75 μM) dropped to two-thirds of that of MDAMB231 (48.19 μM) and was close to that of MCF7 (25.43 μM) (**Table 3**, **Figure 5**). We also tested the sensitivity of AdKu-231 cells in response to the synergistic effect of Tamoxifen and Doxirubicin as compared with MDAMB231, MCF7, and MCF10A (**Figure 5**). Combined use of Tamoxifen and Doxirubicin largely increased cells’ sensitivities. While cancer cells share similar Tamoxifen IC50s which are distinctive from that of normal cells when Tamoxifen was combinatorially used with 10 nm Doxirubicin (lowest tested dose, **Figure 5**), AdKu-231 shares a similar Tamoxifen response curve with MCF7 and MCF10A, which is distinct from that of MDAMB231 under IC50 dose of Doxirubicin (**Figure 5**).

**Table 3:** IC50 of each cell line in response to Tamoxifen, Doxorubicin, or their combination. “IC50-STD” represents the standard deviation of IC50. “AdKu” represents the stable cell line we established with reduced ANLN and increased KDR gene expression.

Cell line	Drug	IC50	IC50-STD
MCF10A	Tamoxifen	16.245	0.3269
MCF7	Tamoxifen	25.4251	1.2181
AdKu	Tamoxifen	29.7514	1.4571
MDAMB231	Tamoxifen	48.1855	2.393
MCF10A	Tamoxifen + 10 nm Doxorubicin	12.9009	0.3451
MCF7	Tamoxifen + 10 nm Doxorubicin	36.3579	2.9832
AdKu	Tamoxifen + 10 nm Doxorubicin	39.0639	2.8264
MDAMB231	Tamoxifen + 10 nm Doxorubicin	42.4501	3.1901
MCF10A	Tamoxifen + 150 nm Doxorubicin	0.3773	0.5591
MCF7	Tamoxifen + 150 nm Doxorubicin	0.6219	1.0033
AdKu	Tamoxifen + 150 nm Doxorubicin	3.3216	2.9751
MDAMB231	Tamoxifen + 150 nm Doxorubicin	39.9377	3.2996
MCF10A	Doxorubicin	154.4694	10.7738
MCF7	Doxorubicin	184.7564	13.1385
AdKu	Doxorubicin	141.9265	20.4547
MDAMB231	Doxorubicin	242.127	20.6849

**Figure 6:**
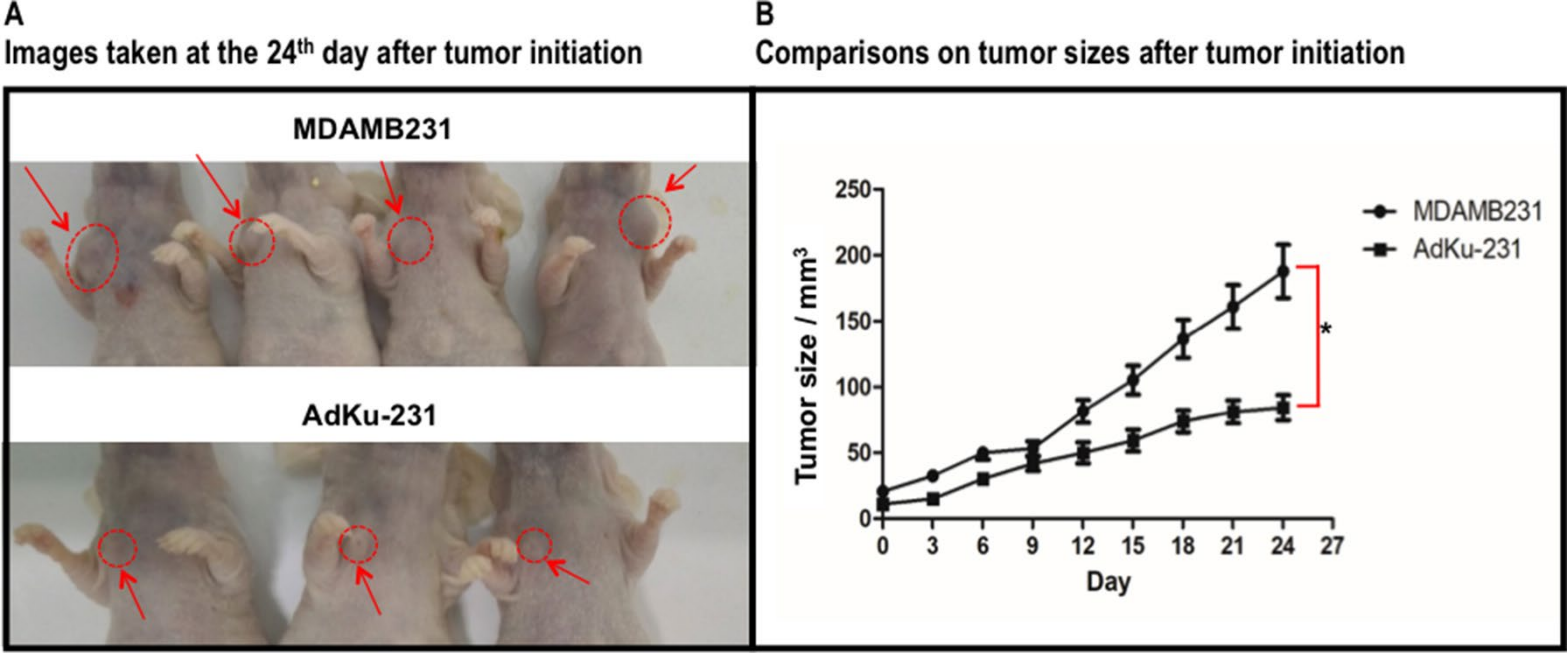
Growth comparison between mouse tumors injected with MDAMB231 and AdKu-231 cells. **(A)** Images taken at the 24th day after tumor initiation. **(B)** Comparisons on tumor sizes after tumor initiation. One mouse injected with AdKu-231 died during the analysis and was dropped out from this study.

*In vivo* study showed slower growth of AdKu-231 cells than MDAMB231 cells (*p* = 0.004, **Figure 6**), which is consistent from what we observed from *in vitro* experiments.

## Discussion

Anillin (encoded by *ANLN*), a relatively poorly understood actin-binding protein involved in cytokinesis and the PI3K/Pten pathway (Suzuki et al., 2005), was found to interact with KDR at both transcriptional and translational levels with opposite clinical implications (**Figure 1**). That is, patients with low *ANLN* and high *KDR* gene expression shared similar favorable clinical outcomes with patients having concomitant high levels of both proteins. Such findings were validated by qPCR and Western blot (**Figure 2**).

These inconsistent clinical associations were driven by ANLN, i.e., low *ANLN* expression at the transcriptional level corresponded to ANLN high expression at the translational level under *KDR* abundance (**Figure 2**). The *p* value and HR were 1.72e−7 and 0.54 for patients with ANLN low expression, which dropped to 7.09e−8 and 0.47, respectively, once KDR was upregulated in addition. This implicates that ANLN drove the main effect of this interaction and KDR has an amplification effect on ANLN functionalities in breast cancer.

*ANLN* mRNA abundance was associated with increased hazard of breast cancer death (**Supplementary Figure 3**). *ANLN* mRNA expression during tumor progression was measured in a diverse spectrum of tumors including breast cancers as well as normal tissues, which showed an increasing trend from the normal to the metastatic state (Wang et al., 2016). Knocking down *ANLN* could significantly decrease the invasiveness and growth of tumor cells (Calvo et al., 2013; Zhang et al., 2018). ANLN was recently proposed as a prognostic biomarker independent of KI-67 (known proliferation marker) and being essential for cell cycle progression in primary breast cancers (Magnusson et al., 2016). These converge to the favorable prognostic value of low *ANLN* mRNA expression among patients and are suggestive of the driving role of *ANLN* in the identified joint prognostic value.

The differential regulatory relationships between ANLN and KDR in different breast cancer cell lines and normal breast cells (**Figure 3**) suggest a potential network rewiring between more and less malignant states in breast cancer cells, which warrants validation at the transcriptional level. Low *ANLN* and high *KDR* gene expression is associated with a favorable clinical outcome, and low *ANLN* is naturally accompanied by decreased *KDR* in malignant tumor cells (**Figure 3**); by externally upregulating *KDR* and downregulating *ANLN* in triple negative cells MDAMB231, we established a cell line sharing similar phenotypical features with luminal breast cancer cells. Cell proliferation, migration, and cancer stem cell assays all suggest that AdKu cells are less malignant than MDAMB231. AdKu cells exhibit similar drug response curve with MCF7 cells under Tamoxifen (Kumar et al., 2018) treatment, suggesting that triple negative cells may be treated using the same strategy as luminal cells if *ANLN* was suppressed and *KDR* was upregulated at the transcriptional level. Indeed, ER, the target of Tamoxifen, was overexpressed on AdKu cells, explaining the demonstrated sensitivity of AdKu cells to Tamoxifen. Triple negative breast cancers are more malignant than the other subtypes and lack effective targeted therapeutic modalities. Triple negative cancers are conventionally treated by chemotherapy or radiotherapy, which are not selective on cancer cells and can considerably reduce the life quality of patients. Poly-ADP ribose polymerase inhibitors target BRCA1-deficient breast cancer cells which cannot represent triple negative breast cancers in general. Our results suggest a novel strategy for triple negative breast cancer control by concomitantly modulating *ANLN* and *KDR* gene expression while administrating Tamoxifen to triple negative patients. That is, by transiting triple negative cancer cells to a less malignant state via concomitantly modulating *ANLN* and *KDR* gene expression, we could obtain desired clinical results using the same strategy as that for luminal cancers. Efforts devoted to cancer state transition, though few, do exist. It was reported that knocking down either *ERN1* or *ALPK1* could push bipotential breast tumor-initiating cells towards the luminal fate (Strietz et al., 2016). Different than that, we focus on the synergistic effects of two pathways (as represented by the identified two genes) on breast cancer state transition, both computationally and experimentally. Importantly, we show direct evidence of combined therapeutic efficacy of the proposed approach, which suggests an emerging cancer therapeutic modality and has profound clinical implications.

## Conclusion

We report that concomitant low *ANLN* and high *KDR* gene expression is associated with favorable breast cancer survival. Externally modulating breast cancer cells towards low *ANLN* and high *KDR* gene expression can transit cells from the triple negative to luminal-like phenotype and sensitize cells to Tamoxifen treatment. This implicates a novel joint therapeutic approach combating against triple negative breast cancers.

## Data Availability

All datasets analyzed for this study are included in the manuscript and the **Supplementary files**.

## Ethics Statement

All animal experiments were performed in accordance with the laboratory animal guidelines and with the approval of the Animal Experimentations Ethics Committee, Jiangnan University.

## Author Contributions

XFD designed, supervised and financed the project, and drafted the paper. XFD and XC conducted computational analysis. YM and DYC conducted the experimental validations. YM and XC prepared the figures. All authors have read and proved the content of the manuscript.

## Funding

This study was supported by the National Science and Technology Major Project of China (grant number: 2018ZX10302205-004-002), Natural Science Foundation of Jiangsu Province (grant number: BK20161130), the Six Talent Peaks Project in Jiangsu Province (grant number: SWYY-128), Postgraduate Education Reform Project of Jiangsu Province, and Research Funds for the Medical School of Jiangnan University ESI special cultivation project (grant number: 1286010241170320). The funders had no role in study design, data collection and analysis, decision to publish, or preparation of the manuscript.

## Conflict of Interest Statement

The authors declare that the research was conducted in the absence of any commercial or financial relationships that could be construed as a potential conflict of interest.
