# Supplementary material for: ANLN and KDR Are Jointly Prognostic of Breast Cancer Survival and Can Be Modulated for Triple Negative Breast Cancer Control
Source: Front Genet. 2019 Oct 4;10:790. doi: 10.3389/fgene.2019.00790 (PMC6788326; doi:10.3389/fgene.2019.00790)
Supplement: Supplementary file 1 [file DataSheet_1.docx]

**Supplementary Table 1. Data sets description.** In ‘Data type’, ‘PEX’ and ‘GEX’ each represents the protein expression and gene expression, respectively. In ‘Analysis’, survival analysis at multiple levels are listed. The total number of samples is shown for each data set, and the number of events in data used for survival analysis is shown in the bracket.

| **Data type** | **PEX** | **GEX** | | | | |
| --- | --- | --- | --- | --- | --- | --- |
| Analysis | TCGA | TCGA | METABRIC | GSE6532 | GSE22220 | GSE24450 |
| PEX survival | 385(43) |  |  |  |  |  |
| GEX survival |  | 514(65) | 1293(295) | 87(28) | 216(82) | 183(39) |

**Supplementary Table 2. Proteins belonging to the PI3K/Pten pathway and subject to the interaction survival anlaysis with KDR.** In 'Source', 'KEGG' means the protein is defined by KEGG and 'REF' means it comes from reference. 'Sig_PEX' and 'Sig_GEX' each represents whether the protein or the encoding gene is significantly associated with breast cancer survival.

| **Proteins** | **Source** | **Sig_PEX** | **Sig_GEX** |
| --- | --- | --- | --- |
| Akt | KEGG | No | No |
| Bcl-2 | KEGG | No | No |
| Bcl-xL | KEGG | No | No |
| Bim | KEGG | No | No |
| Cyclin_D1 | KEGG | No | No |
| Cyclin_E1 | KEGG | No | No |
| p27 | KEGG | No | No |
| eIF4E | KEGG | No | No |
| 4E-BP1 | KEGG | No | No |
| Fibronectin | KEGG | No | No |
| FOXO3a | KEGG | No | No |
| mTOR | KEGG | Yes | No |
| IRS1 | KEGG | No | No |
| KDR (VEGFR2) | KEGG | No | No |
| K-Ras | KEGG | No | No |
| MEK1 | KEGG | No | No |
| ERK2 | KEGG | No | No |
| c-Myc | KEGG | No | No |
| NF-kB-p65_pS536 | KEGG | No | No |
| PDK1_pS241 | KEGG | No | No |
| PI3K-p110-alpha | KEGG | No | No |
| AMPK_alpha | KEGG | No | No |
| PKC-alpha | KEGG | No | No |
| PTEN | KEGG | No | No |
| C-Raf | KEGG | No | No |
| S6 | KEGG | No | No |
| p70S6K | KEGG | No | No |
| LBK1 | KEGG | No | No |
| p53 | KEGG | No | No |
| Tuberin | KEGG | No | No |
| 14-3-3_epsilon | KEGG | No | No |
| CDH1 | REF | No | No |
| Caveolin1 | REF | No | No |
| Claudin7 | REF | No | No |
| ERalpha | REF | No | No |
| ANLN | REF | Yes | Yes |

**Supplementary Table 3. The sgRNA design for the modulation of ANLN and KDR in the experiments.**

| **Type** | **Function** | **Gene** | **Sequence** | **Catalog No.** | **Company** |
| --- | --- | --- | --- | --- | --- |
| sgRNA | enhance | KDR | GTTGTGTGGGGAAATGGGGA | CS30465 | abm |
| sgRNA | enhance | KDR | TGTAAATGGGCTTGGGGAGC | CS30465 | abm |
| sgRNA | knock down | KDR | TGTGGCTCTGCGTGGAGACC | CS30462 | abm |
| sgRNA | knock down | KDR | AGCCTACAAGTGCTTCTACC | CS30462 | abm |
| sgRNA | enhance | ANLN | GCCTCCGCGCTCTAGAGTTT | CS30464 | abm |
| sgRNA | enhance | ANLN | GAGATCGCCGCCGGGTACCC | CS30464 | abm |
| sgRNA | knock down | ANLN | GAAGATTCTCTCGCCTGGCA | CS30463 | abm |
| sgRNA | knock down | ANLN | GCCGAGGCATTTGAAAGCAG | CS30463 | abm |

**Supplementary Table 4. Primers used in qRT-PCR.**

| **Gene** | **Forward primer** | **Reverse primer** |
| --- | --- | --- |
| ANLN | TGTTCTGGACAAGGTCCCCT | ACACCATCTTCGATGCCAGG |
| KDR | ATGCATCCTTGCAGGACCAA | GGTTTCCTGTGATCGTGGGT |
| ER | CAGGCATTCGGTTTGATGAGT | TTGGACGAAGTACAGTTCCCG |
| HER2 | TGTGACTGCCTGTCCCTACAA | CCAGACCATAGCACACTCGG |
| GAPDH | CCCACTCCTCCACCTTTGAC | ATGAGGTCCACCACCCTGTT |

**Supplementary Figure 1. Identified players of the PI3K/Pten pathway according to KEGG.** The PI3K/Pten pathway was retrieved from KEGG, and the 31 proteins with data available in TCGA were highlighted in red.

**
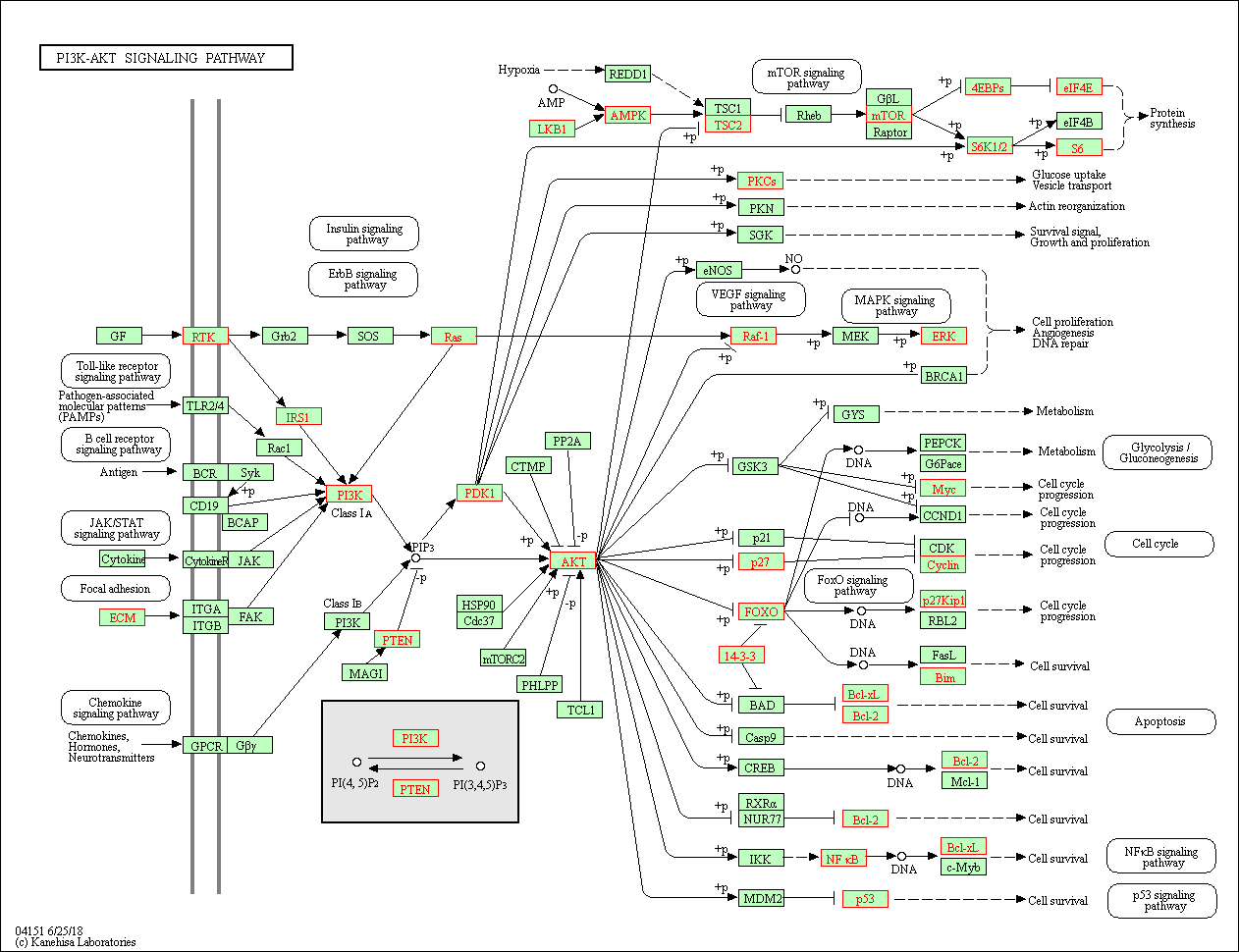
**

**Supplementary Figure 2. Protein expression of ANLN stratified by KDR (A-B) and the main effect of ANLN and KDR (C-D) on patients survival.** The number shown in the bracket is the actual number of samples at the corresponding expression level of ANLN when KDR is high (A) or low (B), or the corresponding expression level of ANLN or KDR among all the samples.


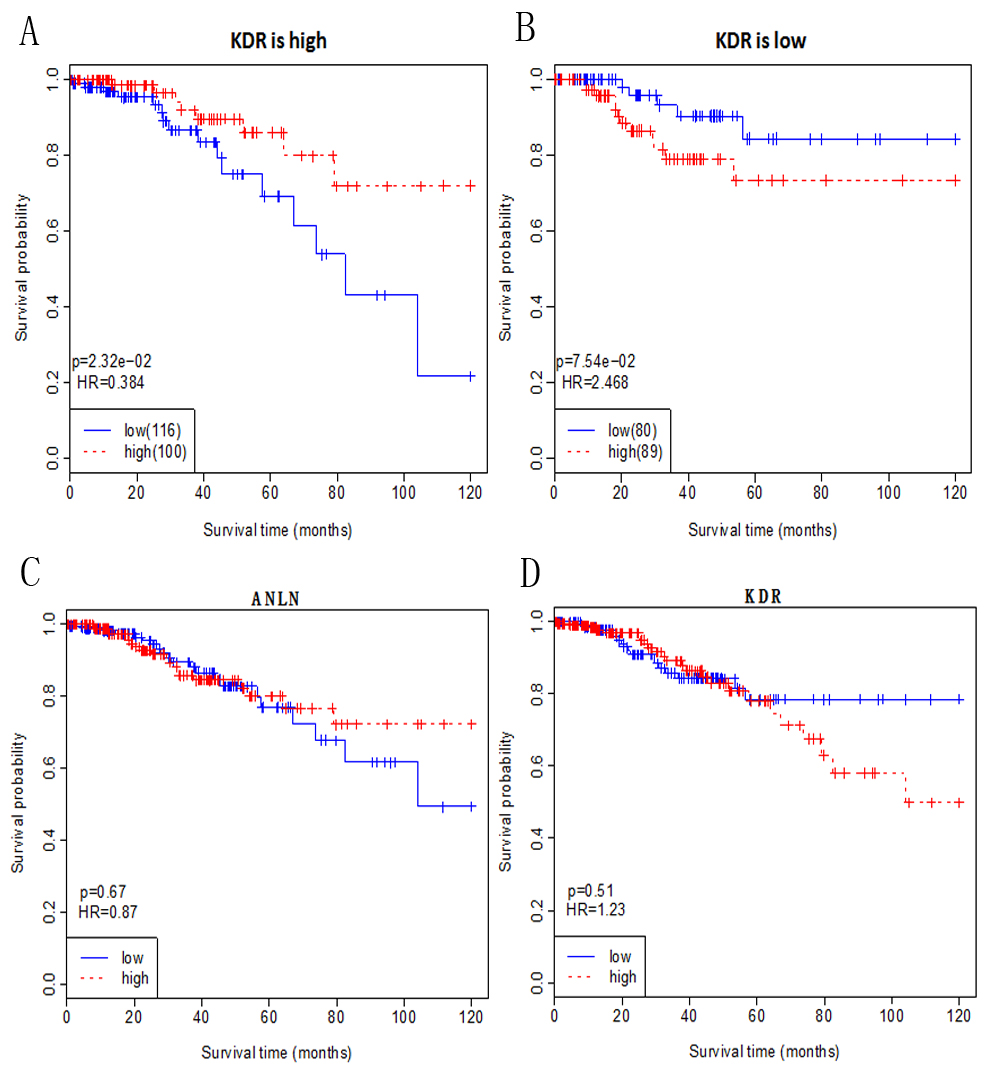


**ANLN**

**ANLN**

**Supplementary Figure 3. Gene expression of ANLN stratified by KDR (A-B, E-F, I-J, M-N, Q-R) and the main effect of ANLN and KDR (C-D, G-H, K-L, O-P, S-T) on patients survival using different data sets.** A-D: METABRIC data set. E-H: TCGA data set. I-L: GSE6532 data set. M-P: GSE22220 data set. Q-T: GSE24450 data set.

**
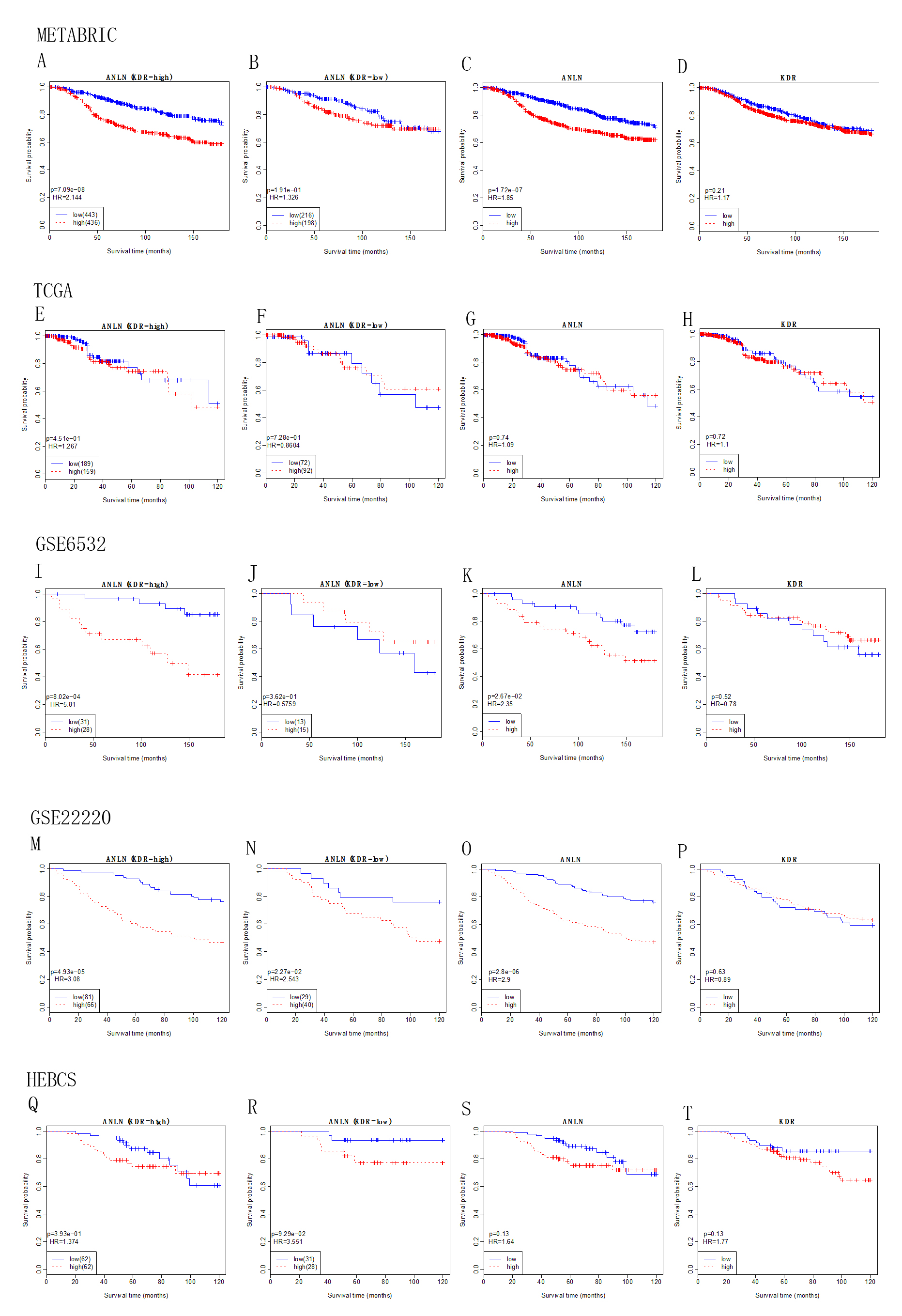
**
